# Assessment of the Release of Vascular Endothelial Growth Factor from 3D-Printed Poly-ε-Caprolactone/Hydroxyapatite/Calcium Sulfate Scaffold with Enhanced Osteogenic Capacity

**DOI:** 10.3390/polym12071455

**Published:** 2020-06-29

**Authors:** Cheng-Yu Chen, Chien-Chang Chen, Chen-Ying Wang, Alvin Kai-Xing Lee, Chun-Liang Yeh, Chun-Pin Lin

**Affiliations:** 1 Graduate Institute of Clinical Dentistry, School of Dentistry, National Taiwan University, Taipei 10617, Taiwan; k55599911@gmail.com (C.-Y.C.); jybean@gmail.com (C.-Y.W.); staryeh0524@gmail.com (C.-L.Y.); 2 3D Printing Medical Research Center, China Medical University Hospital, China Medical University, Taichung 40447, Taiwan; m0975371198@gmail.com (C.-C.C.); Leekaixingalvin@gmail.com (A.K.-X.L.); 3 Section of Periodontics, Department of Dentistry, National Taiwan University Hospital, Taipei 10617, Taiwan; 4 School of Medicine, China Medical University, Taichung 40447, Taiwan; 5 Department of Dentistry, National Taiwan University Hospital, Taipei 10617, Taiwan

**Keywords:** vascular endothelial growth factor, 3D printing, porous scaffold, hydroxyapatite, calcium sulfate, bone regeneration

## Abstract

Vascular endothelial growth factor (VEGF) is one of the most crucial growth factors and an assistant for the adjustment of bone regeneration. In this study, a 3D scaffold is fabricated using the method of fused deposition modeling. Such a fabricated method allows us to fabricate scaffolds with consistent pore sizes, which could promote cellular ingrowth into scaffolds. Therefore, we drafted a plan to accelerate bone regeneration via VEGF released from the hydroxyapatite/calcium sulfate (HACS) scaffold. Herein, HACS will gradually degrade and provide a suitable environment for cell growth and differentiation. In addition, HACS scaffolds have higher mechanical properties and drug release compared with HA scaffolds. The drug release profile of the VEGF-loaded scaffolds showed that VEGF could be loaded and released in a stable manner. Furthermore, initial results showed that VEGF-loaded scaffolds could significantly enhance the proliferation of human mesenchymal stem cells (hMSCs) and human umbilical vein endothelial cells (HUVEC). In addition, angiogenic- and osteogenic-related proteins were substantially increased in the HACS/VEGF group. Moreover, in vivo results revealed that HACS/VEGF improved the regeneration of the rabbit’s femur bone defect, and VEGF loading improved bone tissue regeneration and remineralization after implantation for 8 weeks. All these results strongly imply that the strategy of VEGF loading onto scaffolds could be a potential candidate for future bone tissue engineering.

## 1. Introduction

Bone defects due to trauma, disease or congenital origins are common but they represent a very sophisticated disorder in our current generation which is affecting millions of people worldwide [1]. The most common causes of bone defects are trauma, tumor and iatrogenic wound infections. Furthermore, with an increasingly aging population, cases of bone defects due to chronic degenerative diseases, such as osteoporosis, osteoarthritis and even hyperparathyroidism, have increased gradually over the years. Current treatment options, regardless of medical or surgical methods, are severely limited by local problems such as abnormal fibrosis and lack of vascularization. The latest gold standard for treating bone defects is bone grafting and various types of grafts exist to serve this purpose, including the traditional methods of autograft, allograft and xenograft, as well as the newer method of artificial bioceramics [2]. It is common knowledge that strategies such as allografts or xenografts have lower success rates and much higher rates of rejection as such grafts are of foreign origin and thus can cause serious tissue inflammation, which would eventually lead to tissue failure. Therefore, autografts remain the most widely accepted treatment method for bone defects. However, problems such as a lack of harvesting sites and the need to undergo multiple surgical procedures and risks severely limit the application of autografting in clinical applications. In addition, chronic degenerative diseases are usually systemic, and, thus, in such patients, we are unable to harvest bones from another autologous site. Furthermore, autografts are also greatly limited by the size of the bone defect. Therefore, there is a need to understand the mechanisms of bone regeneration and formation in order to overcome these problems and make a breakthrough [3].

Bioceramics is a common material used for bone and dental applications such as periodontal repair, mandibular reconstruction and even for the fabrication of artificial joints [4]. There are several types of biomaterials, such as hydroxyapatite (HA), calcium sulfate (CS) and calcium silicate, which are commonly used due to their high biocompatibility, bioactivity and low cytotoxicity [4]. In addition, it was also reported that their structures are able to mimic native bones and can effectively help bone repair. For instance, the microstructure of bioceramics allow it to have the ability to promote ossification and neovascularization, and such abilities are critical for osteoinduction, osteoconduction and osseointegration [5]. In recent years, bioceramics combined with three-dimensional (3D) printing is starting to become a popular trend due to its excellent customization, biocompatibility and bioactivity [6]. Such 3D printing technology has been widely applied to bone tissue engineering as it gives us the flexibility to customize personalized grafts according to one’s need quickly and with reproducibility [7,8,9]. Certain parameters are known to be characteristics and properties of scaffolds, such as porosity, pore size and shape, sinter temperature, crystal distribution and phase composition [10,11,12]. It is worth noting that porosity is a critical factor and a uniform porosity was shown to allow good bio-resorption, biodegradation and a stable release rate of ions, thus leading to subsequently enhanced bone regeneration, bone remodeling, angiogenesis and vascularization [13]. The application of 3D printing in medicine thus allows us to fabricate scaffolds with tunable structural characteristics in order to overcome clinical problems [14,15,16]. Recent studies have shown that the modification of bioceramics with natural materials can enhance the biocompatibility and bioactivity of bioceramics, thus making it a better potential candidate as compared to pure bioceramics [17,18]. Several studies have indicated that modification with HA bioceramics composites was able to improve the material’s physicochemical properties, enhancing its mechanical properties and accelerating cellular proliferation and differentiation, thus leading to the promotion of osteoconduction [19,20,21]. HA is a type of calcium phosphate which is known to be the most stable amongst the various calcium phosphates. Its precipitation can form high-density crystallization, with a Ca/P ratio of 1.67. [22]. However, there were no significant results from these initial studies as HA was directly applied to bone defects and live tissues. This occurred during the later stages, when studies began mixing HA into synthesizable materials and fabricating them into scaffolds. Such studies showed enhanced osteogenesis and osteoinduction capabilities, thus laying a foundation for future bone tissue engineering research [23]. On the other hand, calcium sulfate (CS) is also a type of calcium phosphate which is widely used in bone repair and remodeling due to its excellent biodegradability and setting time [24]. Recent novel studies inspired by mussels have shown that growth factors can be attached to scaffolds via a simple immersion technique [15]. Many studies have since then attempted to modify the surface of bioceramic scaffolds in order to enhance the bioactivity of scaffolds [25]. In the previous study, we developed HACS bioceramics with the sustained release of growth factors and this demonstrated a high potential to promote hard tissue regeneration [26].

Vascular endothelial growth factor (also called VEGF-A or VEGF) and its corresponding receptor (VEGFR) have been shown to mediate physiological reactions such as angiogenesis and bone development and they even play a role cancer development [27]. VEGF is a homodimer glycoprotein which belongs to a family of multipotent cytokines including VEGF-A, VEGF-B, VEGF-C, VEGF-D and PIGF. VEGF was originally observed as a growth factor which showed the behaviors of increased vascular permeability and endothelial cell migration and proliferation, which have well-known effects on angiogenic differentiation [28]. Moreover, VEGF was reported to be the most important growth factor in vessel development [7]. It is common knowledge that angiogenesis is essential for the regulation of many physiological functions and even for bone regeneration and ossification [29]. In the process of bone regeneration, VEGF plays a vital role in promoting osteoblast formation and mineralization at the site of the bone defect [30]. In addition, osteoblasts are known to secrete VEGF-A, which has been shown to promote osteoprogenitor cell differentiation and also enhance the secretion of bone-related cytokines such as collagen, Runx2, transforming growth factor (TGF) and alkaline phosphatase (ALP) which allow for increased extracellular matrix (ECM) and bone matrix remodeling [31]. Numerous studies have attempted to combine various types of bioceramics with HA and it has been widely reported that such combinations have several limitations, such as unstable degradation, thus leading to hydroxyapatite agglomeration issues.

Therefore, in this study, our hypothesis was that a combination with CS would make up for the limitations of HA by stabilizing degradation rates and release rates, and thus we attempted to fabricate a hybrid mesoporous bioceramic material made out of HA, CS, and polycaprolactone (PCL). Our study used 3D printing to fabricate porous scaffolds made up of HACS, which was then modified with a VEGF coating. It was shown that these scaffolds were not only able to enhance angiogenesis but also stimulate bone regeneration by enhancing cell proliferation, differentiation and cytokine secretion. This article provides a novel insight into bone tissue engineering by showing the synchronous cooperation of VEGF with bioceramics.

## 2. Results and Discussion

### 2.1. The Characterization of HACS Scaffold

**Figure 1:**
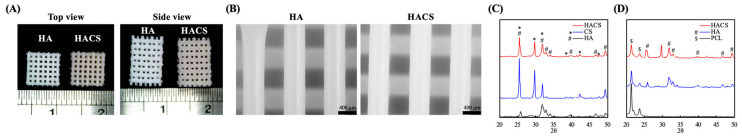
The photographs of (**A**) different view, (**B**) high magnification and (**C**) X-ray diffraction patterns of the hydroxyapatite (HA), calcium sulfate (CS) and HACS powder and (**D**) the 3D printed HA, HACS and PCL scaffolds.

Recently, 3D printing has emerged as a suitable and potential technique to fabricate personalized scaffolds for various clinical conditions. Both hydroxyapatite (HA) and calcium sulfate (CS) are known to be materials with excellent levels of biocompatibility and are widely used in tissue engineering as bone graft substitutes and even as drug carrier devices [32]. HA and CS have been investigated in both in vitro and in vivo studies and initial results have revealed that both HA and CS were able to enhance bone regeneration [33]. Photo images of the scaffolds were taken, and these are shown in Figure 1A. As seen, the scaffold was a 6 mm × 6 mm × 10 mm rectangle and the distance between each layer was 400 µm (Figure 1B). In addition, each strut and pore were uniform and the shearing tears were neat at each end of the strut. The XRD distribution of each type of scaffold is shown in Figure 1C,D. The strongest diffraction peaks of the HACS scaffolds were found at 2θ = 26°, 29° and 32.5°, thus indicating that the crystalline hydroxyapatite was present in the scaffolds. In our previous study, we demonstrated that HA-based scaffolds had peaks at 2θ = 26°, 29° and 32.5°, further confirming the presence of HA in our present scaffold [24]. Furthermore, we also showed that HA-based scaffolds had high biocompatibility and that the pores that were well-interconnected were able to enhance bone and tissue regeneration [34]. In recent years, there have been increasing reports and evidence showing that scaffolds with uniform pores and rough surfaces had enhanced cellular proliferation and differentiation as compared to scaffolds with flat surfaces and no pores [35]. Furthermore, Huang et al. reported that a 3D-printed mesoporous calcium silicate scaffold could enhance the strength and biocompatibility of scaffolds, thus making it more suitable for bone tissue engineering [36]. Similarly, our previous studies demonstrated that porous scaffolds with good connectivity and permeability were able to enhance dental pulp cell proliferation and attachment [36]. In this study, we successfully fabricated porous HACS scaffolds using 3D printing. Results showed that HA was presented in our scaffolds and that scaffolds could be fabricated with the desired structural uniformity and characteristics. Therefore, in consultation with the results from our previous studies and by others, we hypothesized that our HACS scaffold could enhance cellular activities and subsequently enhance bone regeneration as desired.

### 2.2. Properties of Immersed Scaffolds

**Figure 2:**
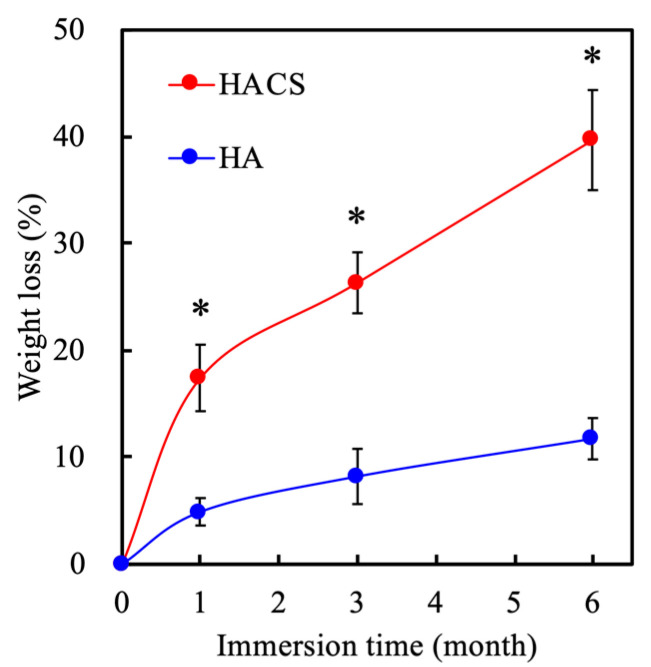
The degradation profile of HA and HACS scaffolds after being immersed in SBF for 6 months. Data are presented as mean ± SEM, *n* = 6 for each group. * *p* < 0.05 compared with HA.

**Figure 3:**
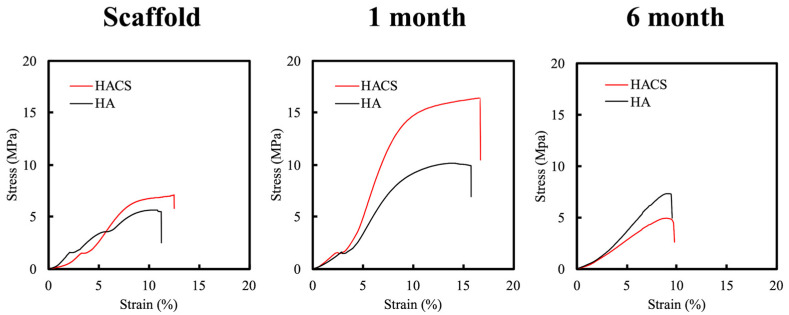
Stress–strain curves of HA and HACS scaffolds before and after immersion in SBF for 1 month and 6 months.

The degradation and the mechanical properties of scaffolds have also been demonstrated as a crucial index for bone and vascular tissue regeneration [35]. The weight loss profile of HACS and HA scaffolds after simulated body fluid (SBF) immersion were determined at different time periods and the results are shown in Figure 2. The weight of the HACS scaffold was reduced gradually during the duration of the soaking, with a 17.44% and 26.34% weight loss at 1 and 3 months, respectively. The HACS scaffold lost a total of 39.70% after 6 months, which was 2.27-fold more than the first month. However, the HA scaffold had just 11.75% of weight loss after 6 months of immersion. This result demonstrated that the HACS scaffold had a faster biodegradation rate as compared to the HA scaffold, thus making it a better candidate for bone tissue engineering, as bone tends to take up to 6 months to heal completely. Scaffolds for bone tissue engineering need to be of a certain mechanical strength as the scaffolds need to withstand a harsh compressive environment after implantation. Figure 3 shows the typical stress−strain curves of the HACS and HA scaffolds at 1 and 6 months. The HACS scaffold was seen to be able to withstand up to approximately 7.06 MPa of stress in its initial stages, which increased to 17 MPa after 1 month. However, the HA scaffolds were only able to withstand up to 5.49 MPa of stress at the initial stage and 9.97 MPa at 1 month. After 6 months of immersion, both the HACS and HA scaffolds had a significant decrease in their mechanical strength, with HACS having 7.2 MPa and HA having 4.9 MPa. Through these results, it can be seen that HACS had better mechanical properties than the HA group, thus making it a better candidate for future bone engineering applications. Furthermore, previous studies have showed a correlation between the degradation and mechanical properties of scaffolds and the biological functions of cells [37]. It was concluded that scaffolds with controllable degradation and better mechanical properties were able to enhance proliferation, vascularization and regeneration. Therefore, it was hypothesized that HACS scaffolds would perform better in enhancing cellular activities as compared to HA scaffolds.

### 2.3. VEGF Release

**Figure 4:**
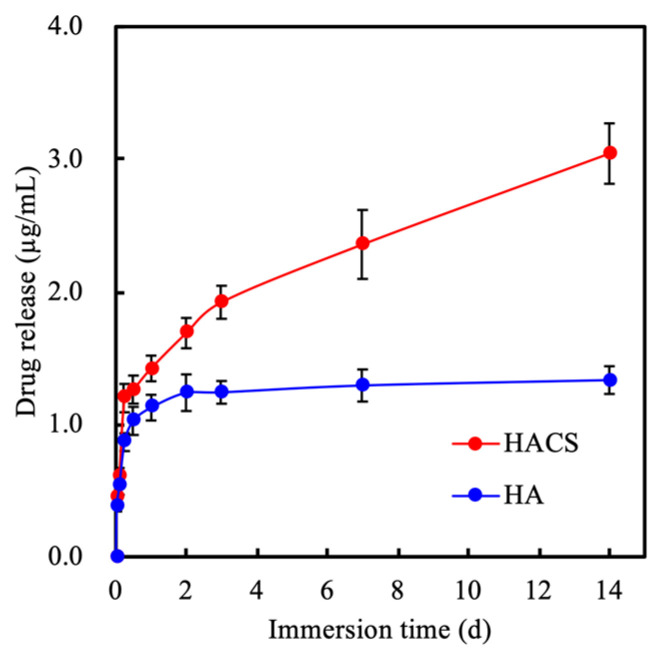
Vascular endothelial growth factor (VEGF) release from HA and HACS scaffolds after immersion in the cultured medium at 37 °C for 14 days.

HACS scaffolds had a higher degradation rate as compared to HA scaffolds, thus making them better candidates for the loading of drugs or growth factors to allow for sustained release over a period of time [38]. Figure 4 shows the cumulative amount of VEGF released from drug-loaded HACS scaffolds in order to assess for their angiogenesis ability. At 1 and 14 days of immersion, the amount of VEGF released from the HACS scaffold was 1.42 μg/mL and 3.04 μg/mL, respectively. Furthermore, it was noted that the VEGF increased in a time-dependent manner over the period of immersion. Recent studies have attempted to load growth factor onto scaffolds so as to enhance wound healing or tissue regeneration [39,40]. According to Li et al., a VEGF-loaded hydroxyapatite–collagen scaffold was able to enhance angiogenesis and osteogenesis in an ischemic limb rat model [31]. Our results revealed that HACS scaffolds had the capability to release VEGF in a time-dependent manner, suggesting that the HACS scaffolds were able to induce neovascularization and thus play a vital role in bone tissue regeneration. In addition, it was hypothesized that the plateau of release from the HA scaffold was due to the slower degradation of HA as compared to HACS. Other reports have been made confirming that drug release could be affected by the rate of degradation. A study by Yusop et al. showed that curcumin release was affected by the addition of iron to scaffolds; as such, a modification was able to enhance the degradation of poly(lactic-co-glycolic acid) scaffolds [41].

### 2.4. Cell Proliferation

**Figure 5:**
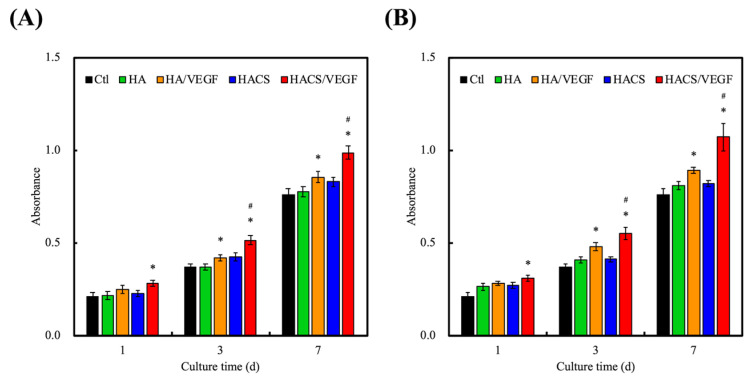
The proliferation of (**A**) human mesenchymal stem cells (hMSCs) and (**B**) human umbilical vein endothelial cells (HUVECs) cultured on different 3D-printed scaffolds. “*” indicates a significant difference (*p* < 0.05) when compared to the scaffold without VEGF. “#” indicates a significant difference (*p* < 0.05) when compared to HA/VEGF.

**Figure 6:**
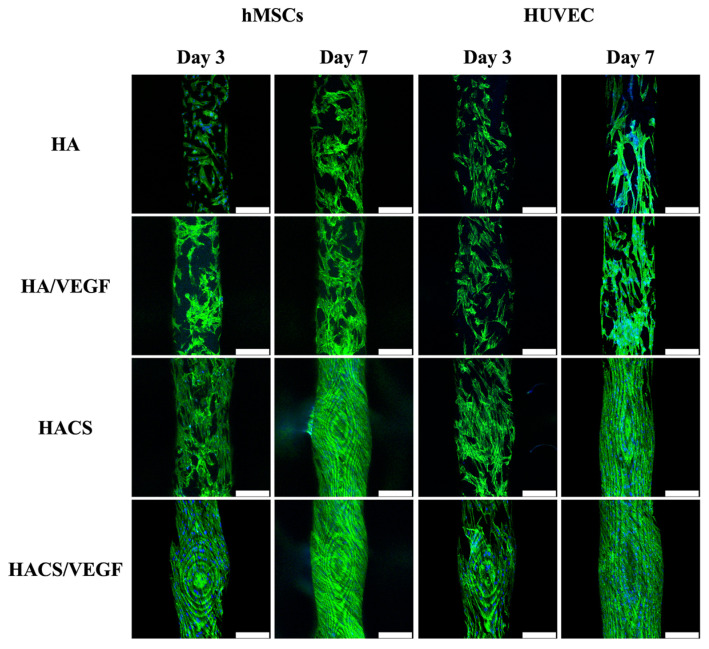
The F-actin filaments (green) and nuclei (blue) staining of hMSCs and HUVECs cultured on the scaffolds for 3 and 7 days. The scale bar is 400 µm.

VEGF was reported to be able to modulate cell function, which includes proliferation, migration and differentiation [42]. For this study, HA and HACS scaffolds were immersed in VEGF solution (0.5 µg/mL) for 12 h to allow for VEGF loading. In addition, the cells were incubated onto the scaffolds for different time intervals. At all time-points, the HACS/VEGF scaffolds had a significantly higher proliferation (*p* < 0.05) of human mesenchymal stem cells (hMSCs) (Figure 5A) and human umbilical vein endothelial cells (HUVECs) (Figure 5B) as compared to the other groups. It was hypothesized that HACS scaffolds were able to release growth factor in a stable manner, thus enhancing cellular growth and proliferation. The VEGF family constitutes the largest sub-group of the growth factor family that was found to play a role in bone development, regeneration and remodeling [43]. Amongst the VEGF family, VEGF-A was the representative for angiogenesis and VEGF-A not only acts as a nutrient for bone cells but also stimulates mineralization and differentiation into osteoblast lineages [44]. Scaffolds carrying VEGF have been shown to promote the secretion of bone-related proteins, such as BMP-2, OPN and OCN, which are all known to be critical components for bone regeneration [45,46]. To further observe for cell morphology, scaffolds were seeded with either hMSCs or HUVECs and cultured on scaffolds for 3 days, and the results are shown in Figure 6. Cellular attachment and proliferation were assessed at 3 and 7 days with F-actin and nuclei imaging. As seen, the cells that were cultured on the HACS scaffold were flattened and had more mitotic spindles and fibers as compared to the control groups. This indicated that HACS/VEGF scaffolds were able to provide a better environment for cells, thus allowing cells to have better attachment and proliferation. The staining assay of MSCs and HUVECs was conducted on different days because of the different cell adhesion abilities. In our results, the two kinds of cells were measured on different days because MSCs need more time to adapt to the scaffold. However, the image of this staining demonstrated that the cell proliferation rate was increasingly elevated as each day passed. This suggests that this scaffold had low cytotoxicity and contributed to cell growth and attachment. It was also important to note that the degree of cellular attachment could be used as a reliable predictor for subsequent cellular behaviors. Reports have been made stating that the cells that were better adhered onto the surfaces of scaffolds had significantly higher proliferation and differentiation as compared to cells which had poor adhesion [47]. Similarly, it could be seen that HACS scaffolds had significantly higher proliferation as compared to HA scaffolds.

### 2.5. Angiogenesis

**Figure 7:**
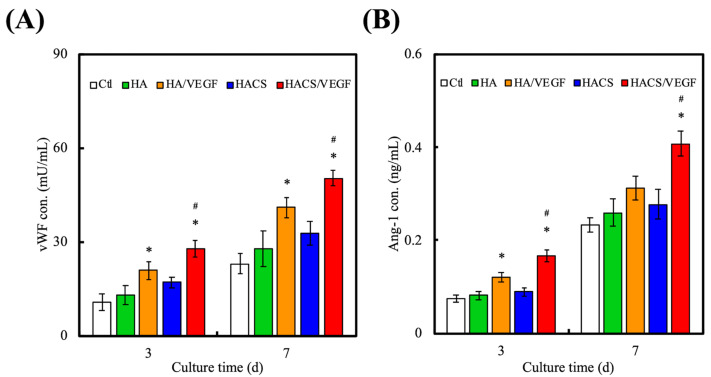
(**A**) The von Willebrand factor (vWF) and (**B**) Angiopoietin-1 (Ang-1) protein expression levels of HUVECs cultured on various scaffolds for 3 and 7 days. “*” indicates a significant difference (*p* < 0.05) when compared to the scaffold without VEGF. “#” indicates a significant difference (*p* < 0.05) when compared to HA/VEGF.

HUVECs were used in this study to verify the angiogenic capability of the HACS/VEGF scaffolds. As seen in Figure 7, the expression of the von Willebrand factor (vWF) and Angiopoietin-1 (Ang-1) were significantly higher for those cells that were cultured on the HACS/VEGF scaffolds. vWF is a complexed protein which is known to regulate homeostasis, angiogenesis and even blood formation. During tissue damage, vWF is known to be able to trap platelets and the surface membrane glycoprotein IIb IIIa to accelerate platelets’ agglutination. Past studies have pointed out that Ang-1 overexpression can increase bone mass and induce expression of bone formation markers (OCN, BMP2 and Runx2) to accelerate new bone formation [48]. Our results demonstrated that, at all time-points, the HACS/VEGF scaffolds had the highest expression of vWF and Ang-1, with the HACS/VEGF scaffolds being 1.3-fold higher than the HA/VEGF scaffolds and three-fold higher than the control groups. It was hypothesized that the increase in activity was due to the stable release of VEGF, thus allowing for enhanced cellular activities [36]. Various studies have demonstrated that VEGF plays a crucial role in mediating angiogenesis, osteogenesis, differentiation, regeneration and bone remodeling [49]. In addition, Choi et al. reported that Ang-1 was critical in the repair of cranial bone defects by activating bone morphogenetic protein 2 and bone-related gene bone sialoprotein, osteocalcin and osterix activity [50]. These results demonstrated that the HACS/VEGF scaffold possesses increased angiogenesis characteristics and is bound to be an indispensable contribution to neovascularization and osteogenesis.

### 2.6. Osteogenesis

**Figure 8:**
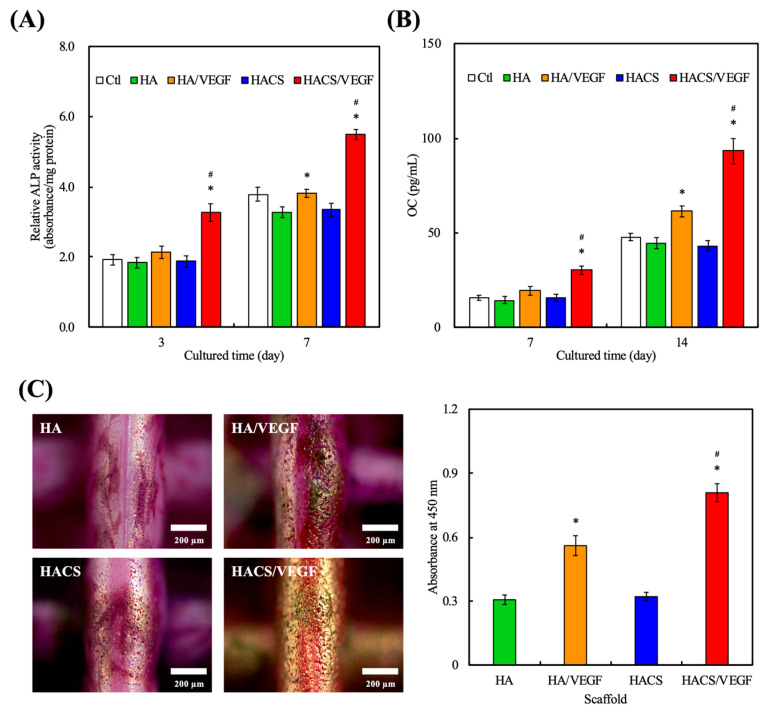
(**A**) The ALP and (**B**) OC protein expression levels of hMSCs cultured on various scaffolds for various time-points. (**C**) Alizarin red S staining and quantification of Ca mineral deposits after culture for 14 days. Data are presented as mean ± SEM, *n* = 3 for each group. “*” indicates a significant difference (*p* < 0.05) when compared to the scaffold without VEGF. “#” indicates a significant difference (*p* < 0.05) when compared to HA/VEGF.

Bone-like mineralized nodules are known to be crucial markers for subsequent mineralization and bone tissue formation. In addition, the extent of nodules is also known to modulate osteoblast differentiation [51]. ALP and osteocalcin (OC) were measured to determine the osteogenic capabilities of the HACS/VEGF scaffolds, and the results are shown in Figure 8. As seen, ALP and OC secretion from HACS/VEGF scaffolds increased in a time-dependent manner. According to the previous study, the differentiation behavior of osteogenic cells was significantly enhanced and the osteogenesis-related gene expressions were up-regulated after being cultured on the VEGF-loading scaffold [52]. The alizarin red S assay was used to confirm the extent of mineralization and it can be seen from Figure 8C that the hMSCs had obviously more mineralization nodules on the HACS/VEGF scaffolds with hMSCs at all time-points as compared to the rest of the groups. After 2 weeks of culture, the nodules on the HACS/VEGF scaffolds were obvious and large enough to be seen with the naked eye. On the other hand, the nodules on the rest of the scaffolds were tiny and sparse. The optical density of alizarin red S was quantified through the ELISA assay and the quantification results were in good agreement with the results presented above. It was reported that a vascularized scaffold with VEGF had an additive effect on cellular differentiation and proliferation as compared with neat bioceramics materials [53]. Therefore, it is important to verify whether the VEGF-loaded scaffold can accelerate bone tissue regeneration in vivo.

### 2.7. In Vivo Bone Regeneration

**Figure 9:**
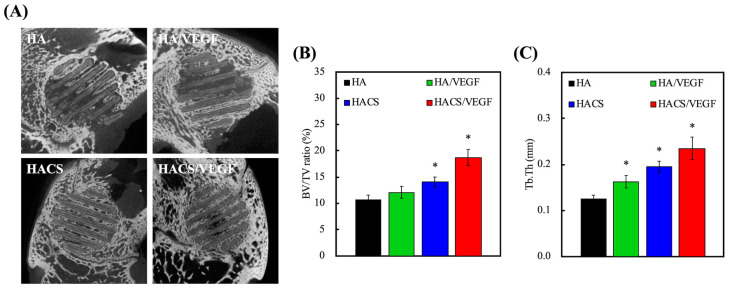
Evaluation of bone formation in vivo. (**A**) Micro-CT images at week 8. (**B**) Micro-CT-quantified histograms of (**C**) bone volume/total volume (BV/TV) and trabecular thickness (Tb.Th). “*” indicate a significant difference (*p* < 0.05) when compared to HA.

To investigate the bone regeneration capabilities of the HACS/VEGF scaffolds, we implanted the scaffolds with a diameter of 6 mm and a height of 6 mm into the femurs of New Zealand white male rabbits. The in vivo study demonstrated that the HACS scaffolds had greater bone regeneration in the femur defects after implantation for 8 weeks, compared with the HA scaffolds. The µCT images (Figure 9A) show that in the HACS scaffold, there was more new bone tissue that could be found and it grew surrounded by the strands of the scaffolds, which served as a template. Moreover, the new bone formation and dense tissue seemed to be obviously improved by the HACS/VEGF scaffold in the defects. The bone volume per tissue volume (BV/TV) ratio of the HACS/VEGF scaffold (18.7% ± 1.5%) was significantly higher compared with the HACS (14.1% ± 0.9%) and HA scaffolds (10.7% ± 0.9%) at 8 weeks, respectively (*p* < 0.05, Figure 9B). Trabecular thickness (Tb.Th) was significantly higher in HACS/VEGF, compared with those of HA and HACS (*p* < 0.05, Figure 9C). The bone-repairing substitutes played an important role in healing these defects in various cases when body environment provisions were inadequate for self-healing, especially in the 3D scaffold [54]. The regeneration of new bone and the bridging of the defect area with HACS/VEGF scaffolds further verified their biocompatibility and osteogenesis nature.

**Figure 10:**
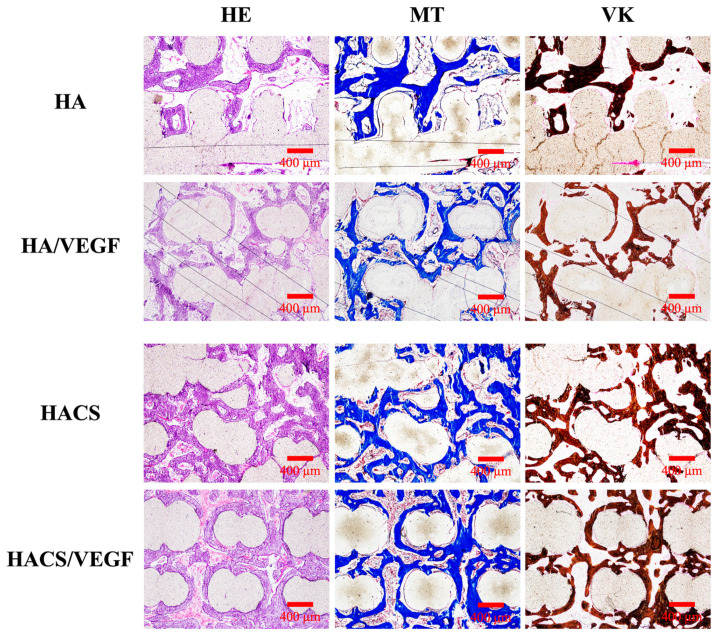
Histological analysis of new bone regeneration around and within the scaffolds in the rabbit femoral defect model. Left: hematoxylin and eosin (HE) stain; middle: Masson’s trichrome (MT) stain; right: von Kossa (VK) stain of regenerated bone mass after 8 weeks of regeneration for in vivo experiment.

**Figure 11:**
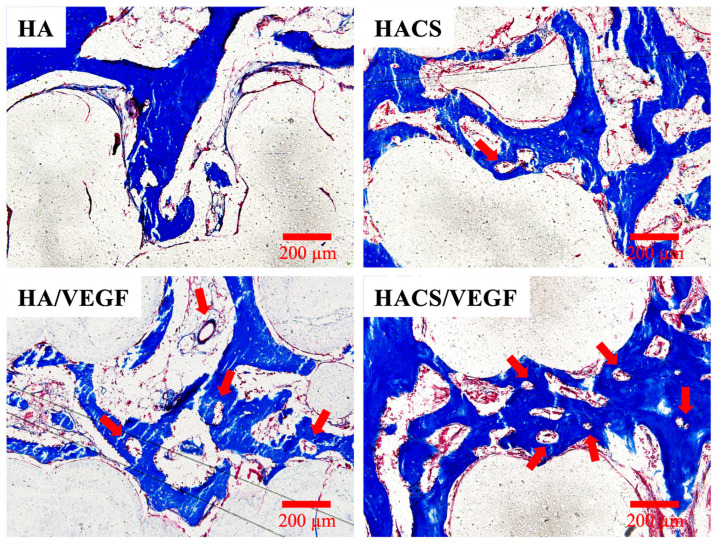
The higher magnification images of MT staining of regenerated bone mass after 8 weeks of regeneration for in vivo experiment. Red arrowheads indicate the blood vessels.

The scaffold and the surrounding tissues were harvested and treated with hematoxylin (HE), Masson’s trichrome (MT) and von Kossa (VK) stains (Figure 10). From the HE staining images, we found that the femur structure and marginal area were the most complete in the HACS/VEGF groups. The MT staining showed the formation of connective tissue, bone collagen and neovascularization in the VEGF-loaded scaffold (Figure 11, red arrows). More newly formed blood vessels could be observed aggregating around the new bone in the HACS/VEGF scaffold. The blood vessels within implants could promote cell proliferation and differentiation that enhance new bone formation and remodeling. In short, the HACS/VEGF scaffolds had better osteoinduction characteristics and these scaffolds were better able to enhance bone regeneration and remodeling as compared with the HA, HA/VEGF and HACS scaffolds. Similarly, it was hypothesized that the HACS/VEGF scaffolds would be able to release VEGF steadily, thus providing extra nutrients to bone cells for subsequent cellular activities. The histological results showed that HACS/VEGF scaffolds were better at enhancing bone regeneration and bone formation in vivo as compared to the rest of the groups. Our study provides a novel insight into how HACS/VEGF scaffolds could increase cell proliferation, osteogenesis and angiogenesis and showed that HACS/VEGF has potential in the treatment of bone defects.

## 3. Materials and Methods

### 3.1. Preparation of HACS/PCL Scaffolds

HA and CS were mixed and milled with a ball in 99% ethanol in a planetary ball mill (Retsch PM-100, Retsch GmbH, Haan, Germany) for 8 h and further dried for 12 h to obtain a raw ceramic powder. X-ray diffraction (XRD; Bruker D8SSS, Karlsruhe, Germany) with a setting of 30 kV, 30 mA and a scanning speed of 1°/min was used to characterize the powder materials. Then, PCL (molecular weight of 43,000–50,000; Polysciences, Warrington, PA, USA) was first heated to 100 °C for 10 min. After this, HACS powder was suspended in absolute alcohol and dripped into the PCL suspension. Finally, the two ratios of HACS/PCL were 50:0:50 and 25:25:50. The mixture was then heated in a 100 °C oven for 1 h. All scaffolds used in this study were fabricated using 3D printing technology via a 3-axis precision positioning system (Bio-Scaffolder 3.1; GeSiM, Grosserkmannsdorf, Germany). Briefly, the dried mixtures were loaded into syringes and dispensed through the 80 °C steel nozzle (400 μm) with an applied pressure of 200–400 kPa and a printing speed of 1.5–2 mm/s. The designs of the scaffolds were as follows: seven 500 µm parallel struts with a 500 µm distance. Then, we used software to quantify the distance between each strut. Each strut had a height of 500 µm, which was quantified by software, and each layer was printed perpendicular to the layer below. The scaffolds were printed into a 6 mm × 6 mm × 10 mm rectangle, as described above.

### 3.2. In Vitro Soaking

The scaffolds were immersed in 37 °C simulated body fluid (SBF) for various durations up to six months. This solution was similar to our native human blood plasma and consisted of 7.9949 g of NaCl, 0.2235 g KCl, 0.147 g K_2_HPO_4_, 0.3528 g NaHCO_3_, 0.071 g Na_2_SO_4_, 0.2775 g CaCl_2_ and 0.305 g MgCl_2_ mixed with 1000 mL of distilled H_2_O. Hydrochloric acid and tris(hydroxymethyl)aminomethane were used to adjust the pH of the SBF solution to 7.4. After immersion for various specified durations, the scaffolds were removed from the SBF and weighed on an analytical balance (TE214S, Sartorius, Goettingen, Germany) scale. The weight of the scaffolds was recorded to analyze for its in vitro degradation profile. The EZ Test machine (Shimadzu, Kyoto, Japan) was used to determine the mechanical properties of each scaffold at a loading rate of 1 mm/min. The average values and standard deviations of Young’s modulus and the maximal compressive strength were evaluated from the recorded stress–strain curves. Six independent tests were done, and the average was recorded.

### 3.3. Cell Culture

In this study, we used human umbilical vein endothelial cells (HUVECs) and mesenchymal stem cells (hMSCs) which were purchased from Sciencell (Sciencell, Carlsbad, CA, USA) and grown in the commercial cell medium (#1001 for HUVECs and #7501 for hMSCs, Sciencell) to passages 3–6, maintained at a 37 °C humidified atmosphere with 5% CO_2_.

### 3.4. VEGF Loading

The scaffolds were immersed in 12.5 µg/mL of VEGF solution (MP Biomedicals, Solon, OH, USA) for the loading of the VEGF [55]. The solution was then placed on an automated shaker for 12 h at room temperature. After this, the VEGF-loaded scaffolds were rinsed with ddH_2_O, freeze-dried and stored in a 4 °C vacuum.

### 3.5. In Vitro VEGF Release Profile

The VEGF release profiles were assessed by soaking the scaffolds in 5 mL of 37 °C Dulbecco’s modified Eagle’s medium (DMEM) for 14 days. The release of VEGF was determined using an enzyme-linked immunosorbent assay kit (Invitrogen, Thermo Fisher Scientific, Carlsbad, CA, USA) according to the manufacturer’s instruction. The concentration was compared to a standard curve and a neat scaffold was used as control. A 96-well spectrophotometer (TECAN Infinite Pro M200, Männedorf, Switzerland) was used to measure for absorbances. All experiments were performed in triplicate.

### 3.6. Cell Adhesion and Proliferation

Prior to cell experiments, all 3D-printed scaffolds were disinfected by soaking them in 75% alcohol followed by 20 min of UV irradiation. HUVECs and hMSCs at a density of 5 × 10^4^ cells/mL were seeded onto the scaffold and cultured at various time-points. The PrestoBlue assay (Invitrogen, Carlsbad, CA, USA) was used to assess for cell viability at pre-determined time points. In brief, 30 µL of PrestoBlue solution and 300 µL of complete growth medium were added into each well and incubated for 30 min. A volume of 100 µL from each well was then transferred to a fresh 96-well ELISA plate and analyzed using a multi-well spectrophotometer at 570 nm with a 600 nm reference wavelength. Cells cultured on culture plates were used as controls (Ctl). All experiments were performed in triplicate.

### 3.7. Fluorescent Staining

After different time-points of culture, the scaffolds were washed with phosphate-buffered saline (PBS) solution, fixed in 4% paraformaldehyde (Sigma-Aldrich, St. Louis, MO, USA) for 15 min and then permeabilized with 0.1% Triton X-100 (Sigma-Aldrich) PBS solution for 15 min at room temperature. The F-actin filaments were then stained with Alexa Fluor 488 Phalloidin (1:300 dilution in PBS, Invitrogen) for 1 h. The nuclei were stained with 300 nM DAPI (Invitrogen) for 30 min. After washing, the morphology was observed using a confocal laser scanning microscope (Leica TCS SP8, Wetzlar, Germany).

### 3.8. Angiogenic and Osteogenic-Related Protein Secretion

The cell was cultured on scaffolds with a commercial cell differentiation medium, such as osteogenic medium from StemPro™ (osteogenesis differentiation kit, Invitrogen), and the angiogenesis starter kit (Invitrogen), for 3 days, in order to assess the levels of alkaline phosphatase (ALP), osteopontin (OPN), osteocalcin (OC), von Willebrand Factor (vWF) and angiopoietin 1 (Ang-1) proteins with an ELISA kit (Invitrogen), according to the manufacturer’s instructions. Protein concentrations were measured by correlation with a standard curve. All experiments were performed in triplicate.

### 3.9. Alizarin Red S Staining

hMSCs were cultured on the scaffolds with an osteogenic differentiation medium for 14 days. This was done in accordance with methods that were previously described [14]. alizarin red S staining was used to determine for calcium deposition. In brief, the specimens were fixed with 4% paraformaldehyde (Sigma-Aldrich) for 15 min and then incubated with 0.5% alizarin red S (Sigma-Aldrich) at a pH of 4.0 for 15 min at room temperature. The cells were then washed with PBS and photographed using a BX53 Olympus fluorescence microscope (Olympus, Tokyo, Japan) at 200× magnification. In addition, the alizarin red S solution was quantified by first adding 20% methanol and 10% acetic acid in water for 15 min. The solution was then transferred to a 96-well plate and the absorbance of alizarin red S was determined using a spectrophotometer with a 450 nm wavelength.

### 3.10. Rabbit Model of Femoral Bone Defects

All the in vivo experimental protocols used in this study were approved by the Animal Experimental Ethics Committee of National Taiwan University. The New Zealand white male rabbits used in this study were 3 months old, with a weight of 1.8–2.0 kg. We used a bone drill to remove bone and create critical-size defects in the femur which were 6 mm in diameter and 6 mm in depth. The scaffolds from the various groups in this study were then implanted at the sites of the critical lesions. The animal experiments were divided into four groups, namely HA, HA/VEGF, HACS and HACS/VEGF. The rabbits were anesthetized with chlorhexidine injections with continuous 5% isoflurane in 100% oxygen, using a gas anesthesia machine (Engler ADS1000, Hialeah, FL, USA). The hair on the hind legs was first shaved with an electric shaver and then disinfected with alcohol and iodine before the dissection of the skin. The muscle fascia was then dissected to expose the femur and special precautions were taken to avoid dissecting excess muscle or causing injury to nerve or blood vessels. The scaffold was then implanted into the defect site, and the wound was closed with sutures and covered with a thick layer of anti-inflammatory ointment. All rabbits fasted for 1 day prior to surgery.

### 3.11. Microcomputed Tomography

Images of all in vivo specimens were taken with a high 360° spatial resolution microcomputed tomography (µCT, SkyScan 1076, SkyScan Inc., Kontich, Belgium) equipped with a 1.4 M X-ray CCD camera. The pre-determined settings were set at: maximum tube current of 0.2 mA, maximum tube voltage of 160 kV, and a 1 mm focus size. For each sample image, a 70-V tube voltage and 70-A current were maintained in the X-ray tube. For the femur scans, slice increments of 30 mm with a total of 400 images were taken. The slices were then analyzed using 3Di-Cat for thresholding to create 3D models for further assessment and quantitative histomorphometric analysis. SkyScan software was used to recognize new bony tissues and to analyze the bone volume per tissue volume (BV/TV) and trabecular thickness (Tb.Th) in the specimens.

### 3.12. Histological Staining

After 8 weeks of culture, the retrieved in vivo samples were fixed in 10% formalin for 48 h, rinsed several times with PBS, embedded in an optimum cutting temperature compound (OCT^®^) (KMA-0100-00A, CellPath Ltd., Newtown, Wales, UK) and sectioned into slices, with 6 mm intervals in between. Longitudinal 6 μm sections were then prepared from each slice using a sawing microtome technique. The sections were then stained with hematoxylin and eosin (H&E) using a modified Masson’s trichrome stain kit (ScyTek Lab., West Logan, UT, USA) and a von Kossa kit (ScyTek), according to manufacturers’ instructions. Collagen was stained blue by trichrome staining while calcified bone was stained red by von Kossa staining, which allowed us to differentiate between the osteoid and calcified tissues. The sections were examined using the BX53 Olympus fluorescence microscope at 50× magnification.

### 3.13. Statistical Analyses

The one-way analysis of variance (ANOVA) was used to determine any statistically significant differences between the means of the experimental groups. Scheffé’s method was used to account for multiple comparisons amongst the group means. All data were considered to be statistically significant with a *p*-value of < 0.05.

## 4. Conclusions

In conclusion, the evidence of the in vitro experiments conducted in the present study suggests that HACS/VEGF scaffolds had good degradation and mechanical strength and were able to enhance ALP, OC, vWF and Ang-1 expression in a time-dependent manner. Furthermore, the evidence of the in vivo results showed that HACS/VEGF had excellent osteoinduction and osteogenesis abilities in rat femur defect models. This article provides an insight into the growth factors derived from the scaffold, which modified cell ability and promoted bone regeneration in the bone defect.
